# Diversity and plant growth promoting properties of rhizobia isolated from root nodules of *Ononis arvensis*

**DOI:** 10.1007/s10482-017-0883-x

**Published:** 2017-05-12

**Authors:** Sylwia Wdowiak-Wróbel, Monika Marek-Kozaczuk, Michał Kalita, Magdalena Karaś, Magdalena Wójcik, Wanda Małek

**Affiliations:** Department of Genetics and Microbiology, Maria Curie -Skłodowska University, Akademicka 19 St., 20-033 Lublin, Poland

**Keywords:** *Ononis arvensis*, Phylogeny, Rhizobia, PGPB

## Abstract

This is the first report describing isolates from root nodules of *Ononis arvensis* (field restharrow). The aim of this investigation was to describe the diversity, phylogeny, and plant growth promoting features of microsymbionts of *O. arvensis*, i.e., a legume plant growing in different places of the southern part of Poland. Twenty-nine bacterial isolates were characterized in terms of their phenotypic properties, genome fingerprinting, and comparative analysis of their 16S rRNA, *nodC* and *acdS* gene sequences. Based on the *nodC* and 16S rRNA gene phylogenies, the *O. arvensis* symbionts were grouped close to bacteria of the genera *Rhizobium* and *Mesorhizobium*, which formed monophyletic clusters. The *acdS* gene sequences of all the isolates tested exhibited the highest similarities to the corresponding gene sequences of genus *Mesorhizobium* strains. The presence of the *acdS* genes in the genomes of rhizobia specific for *O. arvensis* implies that these bacteria may promote the growth and development of their host plant in stress conditions. The isolated bacteria showed a high genomic diversity and, in the BOX-PCR reaction, all of them (except three) exhibited DNA fingerprints specific only for them. Our studies showed that restharrow isolates formed effective symbiotic interactions with their native host (*O. arvensis*) and *Ononis spinosa* but not with *Trifolium repens* and *Medicago sativa* belonging to the same tribe Trifolieae as *Ononis* species and not with *Lotus corniculatus*, representing the tribe Loteae.

## Introduction

The family *Fabaceae* represents a large family of angiosperms. It includes about 750 genera and 19,000 plant species (Graham and Vance 2003; Weir et al. 2004). Within this family, the genus *Ononis* comprises about 70 species occurring in Europe, Asia, and Africa. In Poland, this genus is represented by three species, i.e., *Ononis arvensis*, *Ononis spinosa*, and *Ononis repens*. The latter two species are subject to partial protection in Poland. Plants belonging to *Ononis* genus are perennial herbs and shrubs. Many species of the genus *Ononis* are a source of therapeutic agents such as essential oils, flavonoid glycosides, and tannins. One of the representatives of this genus is *O. arvensis* L. The ingredients of this plant are used in traditional folk medicine for the treatment of various diseases, e.g., those connected with bladder and kidney (Kirmizigul et al. 1997; Suntar et al. 2011; Gampe et al. 2016). Based on the potential value of the *Ononis* species, we decided to collect and characterize 29 strains associated with *O. arvensis*, using different approaches including phenotypic as well as genotypic characteristics for their potential use as PGPB.

Nitrogen is an essential element for the functioning of all living organisms. It is worth noting that nitrogen is also one of most potent plant-growth limiting nutrients (Greenwood 1982). The process of biological nitrogen fixation (BNF), providing nitrogen for soil fertility, is carried out by free-living soil microorganisms and by microorganisms forming symbiotic associations with higher plants. Rhizobial strains are able to form symbiosis with leguminous plants and to convert atmospheric N_2_ into ammonium (NH_4_) in the process of nitrogen fixation, which takes place in special plant organs called root or stem nodules.

Rhizobia represent the group of Alphaproteobacteria and Betaproteobacteria. Already, many genera and species of these bacteria have been described. Alphaproteobacteria comprise microorganisms of the genera *Rhizobium, Mesorhizobium, Ensifer, Azorhizobium, Bradyrhizobium, Methylobacterium, Devosia, Ochrobactrum, Phyllobacterium, Shinella*, and *Microvirga*, while Betaproteobacteria are represented by bacteria of the genera *Burkholderia* and *Cupriavidus* (Wei et al. 2002; Ardley et al. 2012; Peix et al. 2015).

Different types of data are used, i.e., phenotypic, genomic, and phylogenetic, to determine the taxonomic position of bacteria. Phylogenetic studies allow determination of the taxonomic position of bacteria and show their relationship with other groups of microorganisms. Phenotypic analysis is used for preliminary classification of bacteria into the genus as well as identification and differentiation of bacteria based on their metabolic and physiological properties. It also facilitates selection of so-called plant growth promoting bacterial strains that may beneficially influence plant growth and development. For many years, the 16S rRNA gene has been commonly used as a molecular marker in defining the taxonomic position of new prokaryotic isolates. However, due to the high conservation of this molecule, it can be used to determine the genus position than that of the species.

Numerical analysis of phenotypic and genotypic features not only allows preliminary determination of the genus position of microorganisms but also facilitates selection of bacteria that have a positive impact on plant growth and development through different mechanisms such as siderophore production, auxin synthesis (indoleacetic acid—IAA), phosphate solubilization, and 1-aminocyclopropane-1-carboxylate (ACC) deaminase production (Hassan et al. 2015; Anwar et al. 2016). Such plant growth promoting rhizobacteria (PGPR) may be used as plant inocula to improve their development. PGPR include both free-living microorganisms, including cyanobacteria, endophytes colonizing plant tissues, and bacteria that are able to establish symbiotic relationships with plants.

In the recent years, many studies on the diversity of rhizobia have been carried out and many new rhizobial species have been described, including those with plant growth promoting properties (PGPB). However, up to now, there have been no reports about N_2_ fixing microsymbionts of *O. arvensis*. This is the first paper concerning the metabolic, phenotypic, physiological, and plant promoting growth properties of *O. arvensis* nodule isolates, as well as genotypic characteristics, 16S rRNA gene and *nodC* sequence analysis, and the presence and phylogeny of *acdS* genes of these bacteria originating from south-eastern Poland. These studies will help us to identify strains effective in plant growth promotion, which are tolerant to stress conditions and they can be used in the future as a biofertilizer in agriculture.

## Materials and methods

### Strain isolation

Twenty-nine strains were isolated from root nodules of *O. arvensis* (a plant growing in south-eastern Poland) using a standard method described by Gnat et al. (2014). The *O. arvensis* plants were carefully uprooted, the rhizosphere soil was shaken off and roots were washed in sterile water to remove adhering soil. Healthy, pink, and firm nodules from legume roots were taken under consideration. The nodules were surface sterilized with 0.1% HgCl_2_ (w/v) for 1 min, 95% ethanol (v/v) for 1 min, then rinsed several times with sterile water. Suspension were made by crushing single nodules in a sterile petriplate containing a few drops of sterile normal saline. A loopful of suspension was streaked on yeast-mannitol medium (YEM) and incubated at 28º C for 3–5 days. Bacteria isolated from *O. arvensis* root nodules were purified by streaking several times on YEM agar (Gnat et al. 2014). The purified strains were kept in YEM medium at 4 °C as well as at −20 °C in YEM medium with 20% (v/v) glycerol.

The ability of the studied strains to induce nodules on *O. arvensis* roots was confirmed by inoculation of plants with these bacteria.

The strains are in the collection bank of the Department of Genetics and Microbiology, Maria Curie-Skłodowska University.

### Phenotypic properties

All strains used in this study are listed in Table 1. The *O. arvensis* isolates were studied for the Gram-staining reaction, structure, colour of their colony (Gnat et al. 2014), and 126 other phenotypic features. All phenotypic tests were done in triplicate, at least twice.

**Table 1:** *O. arvensis* microsymbionts and reference strains used in this study

Strain	Host plant	Geographic origin	Source
*Ononis arvensis* microsymbionts: (OA) 2, 4, 24, 38, 43, 47, 52, 61, 67, 100, 108, 109, 118, 121, 129, 132, 147, 155, 156, 164, 178, 179, 185, 191, 192, 194, 201, 245, 303	*Ononis arvensis*	Poland	ZGM
*Mesorhizobium amorphae* ICMP15022	*Amorpha fruticosa*	New Zealand	ICMP
*Mesorhizobium albiziae* CCBAU61158	*Albizia kalkora*	China	CCBAU
*Mesorhizobium caragenae* CCBAU11299	*Caragana* spp.	China	CCBAU
*Mesorhizobium chacoense* USDA4963	*Prosopis alba*	Argentina	USDA
*Mesorhizobium mediterraneum* USDA3392	*Cicer arietineum*	Spain	USDA
*Mesorhizobium ciceri* USDA3383	*Cicer arietineum*	Spain	USDA
*Mesorhizobium huakuii* USDA4779	*Astragalus sinicus*	China	USDA
*Mesorhizobium loti* USDA3471	*Lotus corniculatus*	New Zealand	USDA
*Mesorhizobium plurifarium* USDA3707	*Acacia senegal*	Senegal	USDA
*Mesorhizobium septentrionale* SDW018	*Astragalus adsurgens*	China	CCBAU
*Mesorhizobium shangrilense* CCBAU65327	*Caragana bicolor*	China	CCBAU
*Mesorhizobium temperatum* LMG23931	*Astragalus adsurgens*	China	LMG
*Mesorhizobium tianshanense* USDA3592	*Glycyrrhiza pallidiflora*	China	USDA
*Bradyrhizobium elkanii* USDA76	*Glycine max*	USA	USDA
*Bradyrhizobium* sp. (*Lupinus*) USDA3045	*Lupinus* sp.	USA	USDA
*Bradyrhizobium liaoningense* USDA3622	*Glycine* max	USA	USDA
*Bradyrhizobium japonicum* USDA6	*Glycine* max	USA	USDA
*Neorhizobium galegae*			
HAMBI1141	*Galega officinalis*	New Zealand	HAMBI
HAMBI1155	*Galega orientalis*	New Zealand	HAMBI
HAMBI1185	*Galega officinalis*	New Zealand	HAMBI
*Rhizobium leguminosarum*			
bv. *trifolii* 21	*Trifolium* sp.	Poland	ZGM
bv. *trifolii* ANU843	*Trifolium* sp.	Poland	ZGM
bv. *viciae* 1	*Vicia* sp.	Poland	ZGM
bv. *viciae* 2	*Vicia* sp.	Poland	ZGM
bv. *viciae* 33	*Vicia* sp.	Poland	ZGM
bv. *viciae* 36	*Vicia* sp.	Poland	ZGM
bv. *viciae* 3841	*Vicia* sp.	Poland	ZGM
*Rhizobium tropici* OUT21	*Phaseolus* sp.	USA	USDA
*Ensifer fredii*			
USDA1-6	*Glycine* sp.	China	USDA
USDA16-1	*Glycine* sp.	China	USDA
USDA440	*Glycine* sp.	China	USDA
*Ensifer meliloti*			
SU47	*Medicago sativa*	Australia	NZP
11	*Medicago sativa*	Poland	ZGM
13	*Medicago sativa*	Poland	ZGM
L5-30	*Medicago sativa*	Poland	ZGM
L54	*Medicago sativa*	Poland	ZGM
MVII	*Medicago sativa*	Poland	ZGM

*ZGM* Department of Genetics and Microbiology, University of Maria Curie-Skłodowska, Lublin, Poland;* USDA* United States Department of Agriculture, Beltsville, MD, USA;* ATCC* American Type Culture Collection, Rockville, MD;* ICMP* International Collection of Microorganisms from Plants, Landcare Research, Auckland, New Zealand;* LMG* Belgian Coordinated Collections of Microorganisms/LMG Bacteria Collection, Ghent University, Belgium;* NZP* Division of Scientific and Industrial Research, Palmerston North, New Zealand

BS agar medium (Sherwood 1970) with bromothymol blue (0.0025%, w/v) as a pH indicator was used to analyse the ability of *O. arvensis* microsymbionts to use different compounds as a sole carbon source. Mannitol present in the BS medium was replaced with one of the following compounds (1%, w/v): glucose, d-galactose, d-arabinose, d-mannose, d-fructose, d-xylose, d-cellobiose, d-raffinose, d-trehalose, l-arginine, l-lysine, l-alanine, l-asparagine, l-tyrosine, l-glutamine, l-histidine, l-threonine, l-rhamnose, dextrin, glycerol, lectin, salicin, sodium citrate, sodium hippurate, sodium tartrate, maltose, starch, sucrose, inulin, and Tween 20, used as a sole carbon source. YEM containing mannitol as a sole carbon source was used as a positive growth control and the medium without any carbon source served as a negative control.

To assess the utilization of various substrates as a sole nitrogen source, NH_4_Cl, present in the BS medium was replaced with one of the following compounds (1%, w/v): NaNO_3_, l-glutamine, l-tyrosine, l-threonine, l-leucine, dl-valine, l- phenylalanine, l-methionine, l-histidine, l-cysteine, dl-isoleucine, l-lysine, l-alanine, l-aspartate, l-glutamate, l-serine, dl-ornithine, l-arginine, l-proline, and glycine.

Growth characteristics for *O. arvensis* nodule isolates was recorded at different temperatures (4, 6, 28, 37, 45 °C), various pH values (pH 5.0–10.0 at intervals of 1 pH units), and different NaCl concentrations (0.5, 1, 2, and 3% NaCl (w/v) in YEM broth and agar in 48–96 h bacterial culture. The motility of the bacterial strains was determined by spreading the bacteria inoculated in the centre of 0.3% YEM agar after 4–5 days of incubation at 28 °C.

### Intrinsic antibiotic resistance

Antibiotic resistance was assessed by the ability of the bacteria to grow on YEM medium (Gnat et al. 2014) supplemented with different concentrations of the following antibiotics (w/v): streptomycin (1 µg/ml, 5 µg/ml, 10 µg/ml, 20 µg/, 25 µg/ml, 50 µg/ml, 100 µg/ml), neomycin (2.5 µg/ml, 5 µg/ml, 10 µg/ml, 20 µg/ml, 40 µg/ml), chloramphenicol (10 µg/ml, 20 µg/ml, 50 µg/ml, 100 µg/ml, 200 µg/ml), rifampicin (2.5 µg/ml, 5 µg/ml, 10 µg/ml, 20 µg/ml, 40 µg/ml), tetracycline (0.5 µg/ml, 5 µg/ml, 10 µg/ml), ampicillin (5 µg/ml, 10 µg/ml, 20 µg/ml, 50 µg/ml, 100 µg/ml), and clindamycin (500 µg/ml, 1000 µg/ml). The plates were incubated at 28 °C for 72–96 h and antibiotic resistance was evaluated based on bacterial growth.

### Tolerance to dyes

To determine tolerance of bacteria to dyes, they were streaked onto the YEM medium supplemented with the following dyes at different concentrations (%, w/v): auramine (0.0065, 0.013, 0.05, 0.2%); methyl red (0.05, 0.1%); neutral red (0.013, 0.05, 0.1, 0.2%); nigrosine (0.05, 0.1, 0.2, 0.5%); acridine orange (0.0065, 0.013, 0.2%); safranin (0.0065, 0.013, 0.05, 0.2%); and methyl green (0.0065, 0.013%). The plates were incubated at 28 °C for 72–96 h and tolerance to dye was evaluated based on bacterial growth.

### Tolerance to heavy metals

Overnight bacterial cultures were streaked on YEM agar medium plates supplemented with different concentrations of heavy metals (w/v): lead in lead acetate (500 µg/ml, 750 µg/ml), zinc in zinc sulphate (250 µg/ml, 500 µg/ml, 750 µg/ml), and cadmium in cadmium chloride (25 µg/ml, 50 µg/ml and 100 µg/ml) (Wani et al. 2008; Elboutahiri et al. 2010). The plates were incubated at 28 °C for 72–96 h and metal tolerance was evaluated based on bacterial growth.

### Siderophore production

The siderophore production was detected by the chrome azurol sulfonate (CAS) plate assay described by Louden et al. (2011). We used *Ensifer meliloti* SU47 as a positive control because this strain has been described as a producer of siderophores. The negative control was CAS medium without inoculation.

### Other tests

The activity of urease, catalase, galactosidase, gluconidase, nitrate reductase, oxidase, peroxidase, and phosphatase as well as litmus milk reaction were tested (Gnat et al. 2014). Melanin production was determined with the method developed by Cubo et al. (1988). Congo Red absorption was studied in YEM medium with 0.0025% (wt/vol) dye (Wdowiak-Wrobel and Malek 2000) and the precipitation of calcium glycerophosphate was checked according to the Hoffer method (1941). Production of indole in tryptophan broth was studied (Gnat et al. 2014). IAA production was examined with the method described by Wdowiak-Wrobel and Malek (2016). The phosphate solubilizing ability of the studied bacteria was tested on Pikovskaya’s agar (Gupta et al. 2012) containing tricalcium phosphate (TCP) as an insoluble phosphate source. The formed halo zone surrounding the colony revealed phosphate solubilization (Arun and Sridhar 2005).

### Numerical analysis

The phenotypic features of the bacteria were coded in the binary system. The similarity rate of the strains was determined by a simple matching (SM) coefficient and their clustering with the UPGMA method using the software NTSYS package.

### DNA isolation

Total DNA was isolated using the guanidium thiocyanate method described by Pitcher et al. (1989). The DNA concentration and its purity were determined with a spectrophotometer (Bio-Rad, SmartSpec™ 3000).

### rep-PCR analysis

Bacterial DNA fingerprints were obtained with the BOX-PCR method using the primer BOX2AR 5′-CTCCGGCAAGGCGACGCTGAC-3′ (Louws et al. 1994). PCR was performed using a ReadyMix™Taq kit, following manufacturer’s specifications (Sigma-Aldrich), i.e., 3 min at 95 °C, 35 cycles of 1 min at 94 °C, 1 min at 53 °C, 8 min at 65 °C, and finally 16 min at 65 °C. The PCR products were identified by electrophoresis on 1.5% agarose gels in 1× TBE buffer. An analysis of the amplified fragments was carried out using the BIO1D v. 11.10 program (Vilber-Lourmat, France). The strains were grouped by the Nei and Li coefficient (Nei and Li 1979) and the dendrogram was constructed using the UPGMA method.

### 16S rRNA, *nodC*, and *acdS* gene amplification

The 16S rRNA gene was amplified by PCR using two primers fD1 and rD1 according to the procedure described by Weisburg et al. (1991).

The *nodC* gene of the *O. arvensis* isolates was amplified by PCR with primers nodCFu and nodCI using cycle parameters reported by Laguerre et al. (2001).

The *acdS* gene was amplified by PCR using primers acdSF (5′-CAAGCTGCGCAAGCTCGAATA-3′) and acdSR (5′-CATCCCTTGCATCGATTTGC-3′), which were designated by analysis of corresponding sequences of bacterial strains available in the GenBank database. The amplification reaction was performed according to the manufacturer’s description using a 25 μl reaction mixture (Sigma-Aldrich) under the following conditions: initial denaturation for 5 min at 95 °C followed by 30 cycles of 30 s at 95 °C, 30 s at 50 °C, and 1 min at 72 °C and then a final 5-min elongation step at 72 °C.

The amplicons of all the genes were electrophoresed on 1% (w/v) agarose gel and then purified using a Clean-up kit (A&A Biotechnology). Sequencing reactions were performed using the BigDye Terminator Cycle Sequencing Kit (Applied Biosystems, USA). The products obtained were cleaned with an Ex-Terminator kit (A&A Biotechnology) and analysed in an automatic 3500 Genetic Analyzer sequencer (Applied Biosystems). The sequences of 16S rRNA, *nodC*, and *acdS* genes were compared with the sequences available in the GenBank and were aligned using ClustalX2 multiple sequence alignment (Larkin et al. 2007). The sequence similarity rate was determined according to the Kimura two-parameter model (Kimura 1980). Phylogenetic trees of the strains studied were constructed using the neighbour-joining method. Bootstrap analysis was based on 1000 resamplings. The MEGA 4.0 version (Tamura et al. 2007) was used for all gene sequence analyses. The positive control was genomic DNA extracted from *Mesorhizobium loti* USDA3471 and *Rhizobium leguminosarum* bv. *trifolii* ANU843 strains. The negative control was the reaction mixture minus the DNA template (PCR master mix and nuclease-free water instead of DNA).

### Nucleotide sequence accession numbers

The GenBank accession numbers for the 16S rRNA gene sequences of *O. arvensis* microsymbionts OA4, OA118, OA121, OA129, OA132, OA245, and OA191 are KU248351-KU248357. The accession numbers of the *nodC* gene sequences for OA4, OA129, OA132, OA245, OA118, OA191, and OA121 are KU248358-KU28364. The GenBank accession numbers for the *acdS* gene sequences reported in this study (OA121, OA129, OA4, OA118, OA191, OA132, and OA245) are KU248365-KU248371.

### Nodulation tests

Seeds of *Trifolium repens*, *Medicago sativa*, and *Lotus corniculatus* were surface sterilized for 3 min in 3% sodium hypochlorite and next washed several times in sterile distilled water. *O. arvensis* and *O. spinosa* seeds were first immersed in concentrated sulfuric acid for 20 min and afterwards washed in sterile distilled water. All seeds were germinated on sterilized water-agar (0.8%) and the seedlings obtained were transferred (one per tube) on sterile nitrogen-free medium slants (Gnat et al. 2015). Three days later, the seedlings rootlets were inoculated with ~10^8^ bacterial cells. The plants were grown for 6–7 weeks in a greenhouse under natural light supplemented with artificial light (200 μE m^−2^ s^−1^, 14 h day/10 h night, at 24/19 °C). Ten tubes were used for each isolate. Non-inoculated plants were used as negative controls. Symbiotic effectivity of rhizobia was estimated based on their nodule formation and the dry weight of shoots of inoculated plants compared to the control.

## Results

### Phenotypic analysis

The bacteria studied were isolated from *O. arvensis* root nodules. In total, 29 isolates were confirmed to be rhizobia by the authentication test with their native host plant (data not shown). All these bacteria formed effective symbiosis with their native host plant (data not presented). They were Gram-negative, rod-shaped bacteria that formed, circular, convex and colourless colonies, 2–3 mm in diameter, on YEM agar after 2–4 days of growth at 28 °C.

The bacteria grew at temperatures from 4 to 37 °C and the optimum temperature for their growth was 28 °C. The pH range of the *O. arvensis* symbionts was from 5 to 10, except for 3 isolates (OA121, OA156 and OA245) that did not grow at pH 10. None of the *O. arvensis* isolates demonstrated an ability to grow at pH 4. The strains exhibited good tolerance to salinity. All of them grew in the presence of 0.5% NaCl in the medium, and thirteen of them grew at 3% NaCl.

It was observed that the isolates studied were able to metabolize a wide range of substrates as a sole carbon source. All strains used 19 from the 30 compounds studied. Eleven carbon sources were used by 87–96% of the nodule isolates. Three of the 16 compounds tested as a sole N source for *O. arvensis* microsymbionts, i.e., methionine, isoleucine, and proline, were utilized by all strains. None of the tested strains was able to utilise histidine, valine, and glycine (Table 2).

**Table 2:** Characteristics of *O. arvensis* strains and related *Mesorhizobium* and *Rhizobium* species

Characteristics	*Ononis arvensis* microsymbionts (n = 29)	*M. loti* NZP 2213^T^	*M. ciceri* USDA 3383^T^	*M. tarimense* CCBAU 83306^T^	*R. leguminosarum* USDA 2370^T^	*R. pisi* DSM 30132^T^	*R. fabae* LMG 23997^T^
Utilization of compounds as sole carbon sources							
Raffinose	+26	+	−	−	+	+	+
Sodium citrate	+5	−	−	−	−	−	+
Dulcitol	+27	+	+	−	+	+	+
Inulin	+	+	−	−	+	−	−
l-Lysine	+21	+	−	−	+	−	nd
d-Mannose	+	+	+	−	+	+	+
Salicin	+28	−	−	−	+	+	+
Starch	+11	+	−	−	+	+	−
l-Tyrosine	+14	+	−	nd	−	nd	−
l-Arginine	+8	+	−	+	−	−	+
Utilization of compounds as nitrogen sole sources							
l-Alanine	+28	−	+	−	+	−	+
l-Glycine	−	−	−	−	−	nd	+
l-Valine	−	−	−	−	−	−	+
l-Phenylalanine	+10	+	+	−	nd	nd	+
Tolerance to pH							
4	−	±	−	−	−	−	−
5	+	+	+	−	−	+	+
Tolerance to NaCl (%)							
1	+26	+	+	−	−	+	+
2	+14	−	+	−	−	−	+
Antibiotic resistance (μg ml^−1^)							
Chloramphenicol 20	+22	−	+	−	+	nd	+
Ampillicin 50	+18	+	−	−	−	nd	−
Neomycin 20	+20	−	−	+	+	+	+
Tetracycline 30	−	−	−	+	−	−	−
Streptomycin 50	+12	+	+	−	+	nd	+

Strains: *Mesorhizobium loti* NZP 2213^T^ (Jarvis et al. 1982, 1997), *Mesorhizobium ciceri* USDA 3383^T^ (Nour et al. 1994), *Mesorhizobium tarimense* CCBAU 83306^T^ (Han et al. 2008), *Rhizobium leguminosarum* USDA 2370^T^ (Ramírez-Bahena et al. 2008), *Rhizobium pisi* DSM 30132^T^ (Ramírez-Bahena et al. 2008), *Rhizobium fabae* LMG 23997^T^ (Tian et al. 2008)

+ positive; ± weakly positive; − negative;* nd* no data available

The analysis of the intrinsic bacterial resistance to antibiotics showed that the bacteria were generally resistant to the antibiotics used, except tetracycline, which inhibited rhizobial growth already at 5 µg/ml in the medium (Table 2).

The results obtained showed that all isolates were urease, nitrate reductase, and beta-d-glucosidase (esculinase) positive and absorbed Congo Red from the agar medium. Most of the tested isolates were phosphatase (72%), oxidase (65%), and catalase (58%) positive, produced IAA (72%), and were capable of phosphate solubilization (96%), calcium glycerophosphate precipitation (68%), and melanin production (44%).

### Numerical analysis

**Fig. 1:**
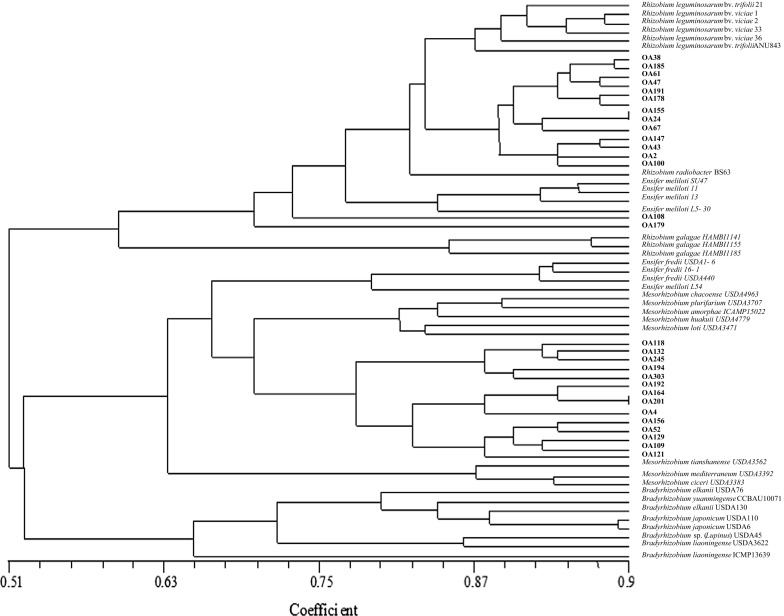
Dendrogram highlighting the phenotypic similarities among rhizobia specific for *O. arvensis* compared with reference species using Jaccard’s similarity coefficients (UPGMA) method

The numerical analysis of 126 physiological and biochemical properties of *O. arvensis* microsymbionts showed their wide phenotypic diversity (Fig. 1). The dendrogram constructed based on this analysis placed the bacteria studied and reference strains in two main clusters at a 51% similarity level (Fig. 1). One cluster contained 15 rhizobial strains specific for *O. arvensis* and 13 reference strains of the genera *Rhizobium* and *Ensifer*. The second cluster included 14 *O. arvensis* symbionts and 11 reference strains of the genus *Mesorhizobium* (Fig. 1).

### Tolerance to heavy metal

The 29 *O. arvensis* microsymbionts were tested for their ability to tolerate various concentrations of heavy metals (cadmium, lead, and zinc) using the streak plate technique. Generally, the strains showed a varied level of tolerance to the heavy metals examined. The *O. arvensis* nodule isolates showed the greatest intrinsic sensitivity to cadmium. Most of them grew in the presence of 50 µg of cadmium per ml of the medium and 20% tolerated even 100 µg of cadmium per ml of medium. Seven of them (OA47, OA61, OA67, OA100, OA108, OA155, OA303) were highly resistant to the three heavy metals studied (cadmium, lead, and zinc).

### Siderophore production

Only six (OA2, OA108, OA109, OA118, OA147, OA43) of the 29 *O. arvensis* microsymbionts produced siderophores, which was demonstrated by formation of orange halos surrounding the spots with bacterial growth on the CAS agar medium.

### BOX-PCR genomic fingerprints

**Fig. 2:**
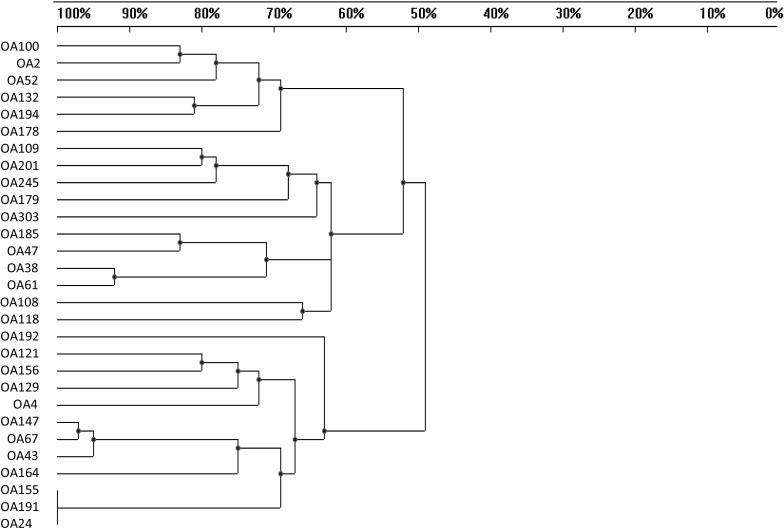
Similarity UPGMA tree of BOX-PCR patterns of 29 *O. arvensis* microsymbionts

Purified total DNAs from the 29 *O. arvensis* microsymbionts were used as templates for PCR with the BOX primer to generate bacterial genomic fingerprints (Fig. 2). In the phylogram based on DNA profiles, the *O. arvensis* microsymbionts were grouped into two clusters at 49% DNA pattern similarity. The bacteria specific for *O. arvensis* showed 27 unique fingerprinting types specific to each strain. Only three strains, i.e., OA24, OA155, and OA191, showed identical DNA fingerprinting patterns.

In total, 593 DNA fragments ranging in size from 593 bp to 9392 bp were obtained using the amplification reaction with the BOX primer and used for construction of a dendrogram by the BIO1D v. 11.10 (Vilber-Lourmat) program (Fig. 2). This analysis showed a high level of genomic diversity among the *O. arvensis* rhizobia studied. The DNA pattern similarity of these bacteria determined by the BOX-PCR method was in the range from 49 to 100% (Fig. 2).

### 16S rRNA gene sequence analysis

**Fig. 3:**
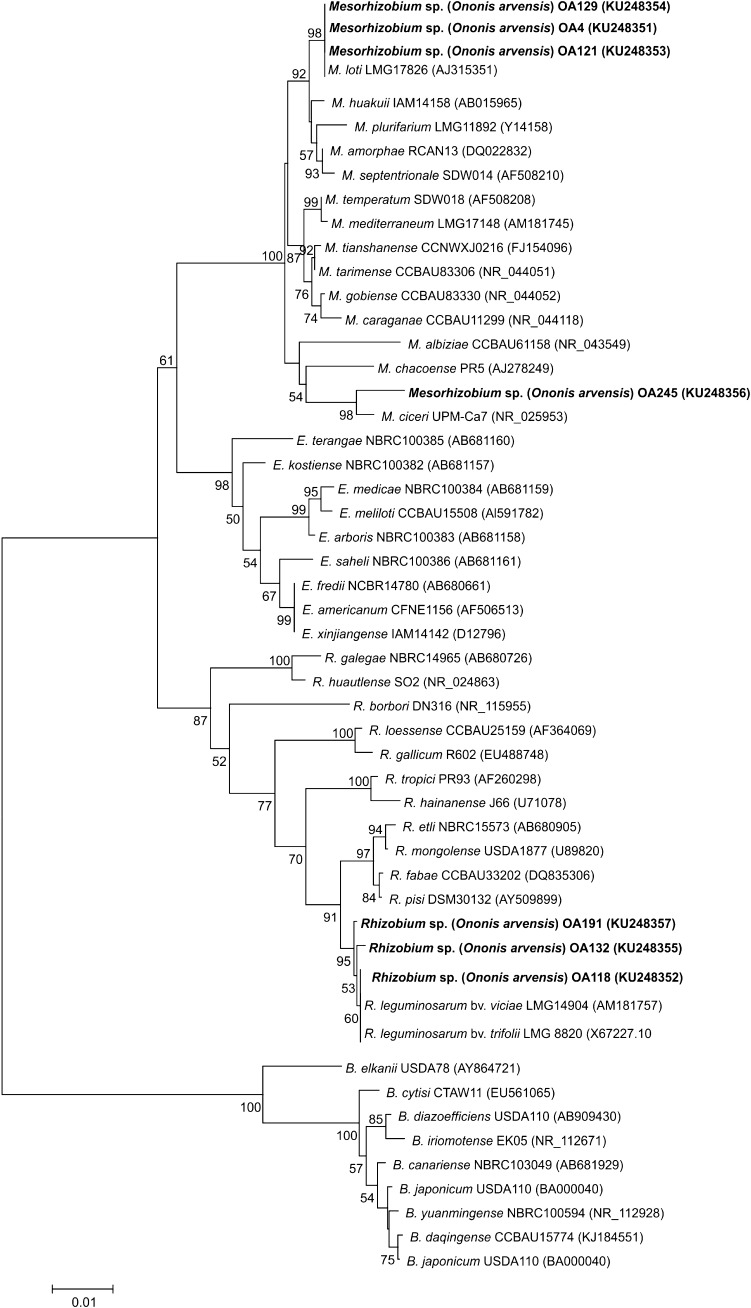
Phylogenetic tree based on partial sequences of the 16S rRNA gene of isolates specific for *O. arvensis* and other rhizobia. The tree was constructed using the neighbour-joining method. Bootstrap values are indicated at the nodes (only values greater than 50% are shown). The *scale bar* at the bottom left represents 0.01 nucleotide substitution per site

In the phylogenetic studies, seven strains representing two groups of *O. arvensis* symbionts identified by the BOX-PCR analysis were used. The comparative 16S rRNA gene sequence analysis of these bacteria (with 92–99% 16S rRNA gene sequence similarity to each other) and 46 reference rhizobia representing different genera and species of nodule bacteria showed that the isolates studied belong to two genera, i.e., *Mesorhizobium* and *Rhizobium* (Fig. 3). In the 16S rRNA gene phylogram, three isolates, OA118, OA132, and OA191, were placed close to the *R. leguminosarum* strains (98–99% sequence similarity) (Fig. 3). The other four isolates, OA4, OA121, OA129, and OA245, were grouped with the genus *Mesorhizobium* strains in one monophyletic cluster with a bootstrap support of 100% (Fig. 3). Three of them (OA4, OA121, and OA129) were close phylogenetic neighbours of *M. loti* strain NZP 2213 (97–100% sequence similarity). Strain OA245 formed a clearly separate lineage together with *Mesorhizobium ciceri* strain UPM-Ca7 (98% sequence similarity).

### *nodC* gene analysis

**Fig. 4:**
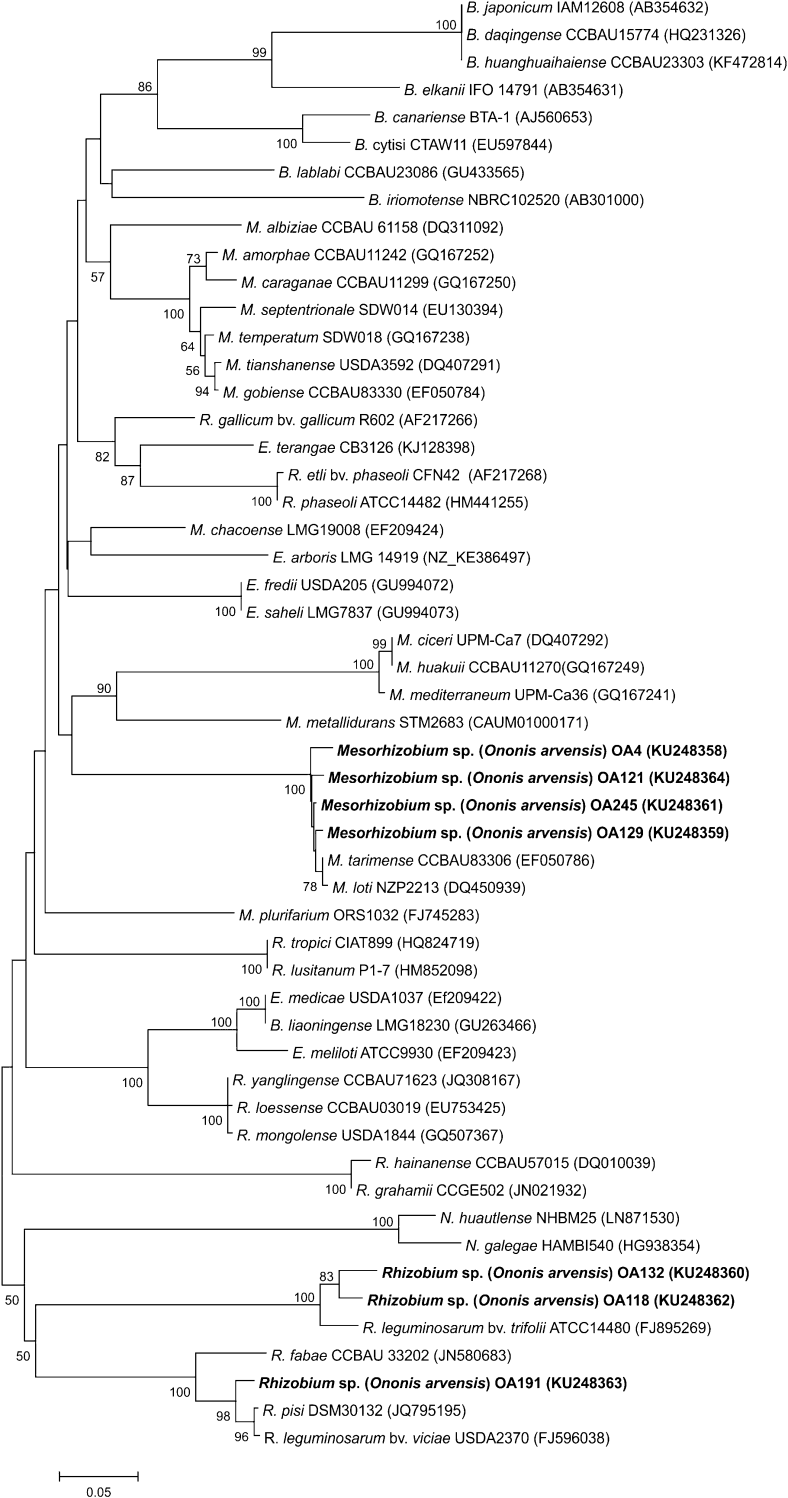
Phylogenetic tree derived from *nodC* gene sequences data. The tree was constructed using the neighbour-joining method. Bootstrap values are indicated at the nodes (only values greater than 50% are shown). The scale bar indicates 0.05 nucleotide substitution per site

The phylogenetic tree built with the partial sequences of *nodC* genes split *O. arvensis* microsymbionts that showed from 69 to 99% similarity to each other into three clades (Fig. 4). The first clade was composed of four *O. arvensis* rhizobia (OA4, OA121, OA129, OA245) as well as *M. loti* NZP2213 and *M. tarimense* CCBAU83306 strains as the close neighbours (98–99% sequence similarity) (Fig. 4). The second one, supported by 100% bootstrap, comprised the OA191 strain, *Rhizobium pisi* DSM30132 (98% *nodC* gene similarity), and *R. leguminosarum* bv. *viciae* USDA 2370 (97% sequence similarity). The two other strains, i.e., OA118 and OA132, were close phylogenetic neighbours of *R. leguminosarum* bv. *trifolii* ATCC14480 and exhibited 94 and 96% *nodC* gene sequence similarities with this strain, respectively.

### *acdS* gene analysis

**Fig. 5:**
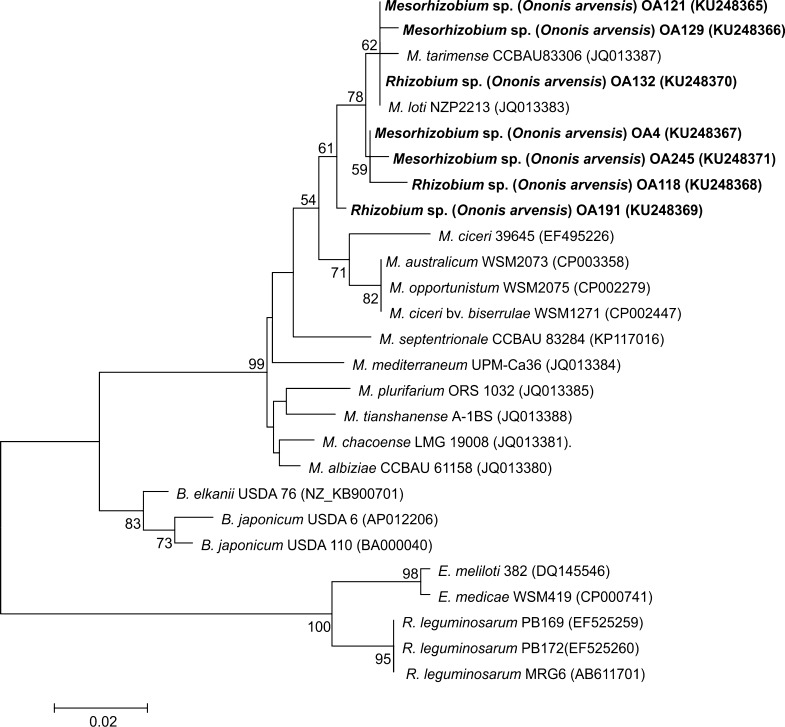
Neighbour-joining *acdS* phylogenetic tree of *O. arvensis* symbionts and rhizobial reference strains. Bootstrap values are indicated at the nodes (only values greater than 50% are shown). The scale bar indicates the number of substitutions per site

The nucleotide sequences of the *acdS* genes of seven *O. arvensis* nodule isolates, OA4, OA118, OA121, OA129, OA132, OA191, and OA245, were compared to those from the GenBank sequence database (Fig. 5). All the strains studied, with 91–98% *acdS* sequence similarities to each other, formed a common cluster with *M. loti* NZP2213 and *M. tarimense* CCBAU83306 as close phylogenetic neighbour (91–99% sequence similarity) and a larger cluster with the other *Mesorhizobium* species included in the analysis (80–99% *acdS* gene sequence similarity) (Fig. 5). The *acdS* sequence similarities of *O. arvensis* rhizobia and *Bradyrhizobium* sp., *Ensifer* sp., *Rhizobium* sp., and *Azorhizobium caulinodans* were substantially lower, i.e., 65–76%, 61–66%, 77–81%, and 72–75%, respectively.

### Nodulation tests

29 isolates from *O. arvensis* nodules, identified on the basis of phenotypic properties and 16S rRNA gene sequence analysis as members of the genus *Mesorhizobium* and *Rhizobium,* were studied for host plant specificity and nitrogen-fixing ability. All the studied isolates were able to induce nodules and fix nitrogen in the symbiotic interaction with their native host plant, as shown by the formation of pink nodules (2–4 per plant) as well as the dark green colour and the dry weight of aerial parts of the inoculated plants, compared with the noninoculated ones. The shoot dry weight of *O. arvensis* inoculated with the studied isolates was 1.85–2.5-fold higher than that of non-inoculated plants.

The nodule inducing and nitrogen fixing abilities of restharrow symbionts were also checked on selected fabacean species that represented hosts of the close relatives of the studied isolates, i.e., *O. spinosa*, *T. repens*, *M. sativa*, and *L. corniculatus*. The nodulation test indicated that 55% of these bacteria nodulated *O. spinosa* and formed pink nodules (1–2 per plant) on this plant; however, rhizobia in this symbiotic association were less effective in N_2_ fixation than in the interaction with *O. arvensis*. Six weeks after inoculation of *O. spinosa* with the tested bacteria, the shoot dry weight of these plants was 1.4–2.2-fold higher compared to the noninoculated plants. None of our isolates nodulated *T. repens*, *M. sativa*, and *L. corniculatus*.

In contrast to the narrow host range of *O. arvensis* symbionts, their native host (restharrow) exhibited (in laboratory plant tests) a wide range of promiscuity and entered mutualistic interactions with *M. loti, Mesorhizobium huakuii, M. ciceri, Mesorhizobium septentrionale, Mesorhizobium albiziae, Mesorhizobium caraganae, Rhizobium etli, Rhizobium tropici*, and *R. leguminosarum* bv. v*iciae*, but not with *Bradyrhizobium, Neorhizobium* and *Ensifer* strains.

## Discussion

Currently, polyphasic taxonomy based on different types of data (phenotypic, genomic, phylogenetic) is used in defining the taxonomic position of microorganisms (Vandamme et al. 1996; Young and Haukka 1996; Niemann et al. 1997). In this study, using the polyphasic approach, we characterized 29 strains isolated from root nodules of *O. arvensis* growing in the south-eastern part of Poland.

Up to now, the phylogenetic position and symbiotic properties of field restharrow microsymbionts have not been characterized and described. The isolates studied have an ability to utilize a wide range of various compounds as sole carbon and nitrogen sources. It was also noted that all of them were fast growers, produced acid on the yeast extract mannitol medium, and formed colonies of 2–3 mm in diameter after 2–3 days of growth at 28 °C on this agar. The capability of acidification of the medium with mannitol is characteristic for fast-growing bacteria, which are also able to utilize different disaccharides. The fast growing rhizobia mainly include nodule bacteria establishing symbiosis with e.g., alfalfa and clover, i.e., plants which belong to the same tribe as the host of the studied isolates. In contrast to fast growing rhizobia, slow growing nodule bacteria cause alkalization of YEM medium and are represented by soybean and cowpea rhizobia (Boakye et al. 2016).

The numerical analysis of the phenotypic properties showed that the *O. arvensis* symbionts are representative of bacteria of the genera *Rhizobium, Ensifer*, and *Mesorhizobium*.

The analysis of the plant growth promoting features of the *O. arvensis* microsymbionts showed that 100% of them were able to produce urease and had phosphate-solubilizing activity. More than 93% of these bacteria (27 strains) produced IAA acid. Research conducted by Antoun et al. (1998) showed that many *Bradyrhizobium* sp. and *Rhizobium* sp. strains produced siderophores and IAA and solubilized phosphate. IAA production by *Rhizobium* sp. strains has also been described by some other authors (Badenoch-Jones et al. 1983; Spaepen and Vanderleyden 2011) and it has been shown that auxin balance in the plant is important for proper nodule organogenesis and for many processes such as division and differentiation of plant cells and formation of vascular bundles (Mathesius et al. 1998).

It is known that the ability of rhizospheric bacteria to synthesize siderophores, antibiotics, HCN, and enzymes such as, e.g., ACC deaminase, phosphatase, chitinase, and urease, is important in the battle against plant root pathogens (Nagarajkumar et al. 2004). Such bacteria are good candidates for plant growth promotion and have a beneficial effect on plant development. It was noted that ACC deaminase synthesized by rhizospheric and endosymbiotic bacteria frequently serves to overcome ethylene stress in plants The presence of active ACC deaminase has been described previously in rhizobial species such as *M. loti, R. leguminosarum* bv. *viciae,* and *Rhizobium hedysari* (Ma et al. 2003; Hao et al. 2011; Gopalakrishnan et al. 2015).

Production of siderophores was detected in the *O. arvensis* microsymbionts (34.4%). The ability to produce siderophores has also been described for *Rhizobium leguminosarium* bv. *viciae*, *Rhizobium leguminosarium* bv. *trifolii*, *E. meliloti, Bradyrhizobium japonicum*, and *M. ciceri* (Guerinot et al. 1990; Reigh and O’Connell 1993; Raychaudhuri et al. 2005). The symbiotic rhizobium-legume plant interaction is iron-dependent, since the presence of this element is necessary for the functioning of some enzymes such as leghemoglobin, nitrogenase, and ferredoxin. Studies have shown that siderophore-producing bacteria positively influence plant growth and the efficiency of host plant nodulation (de Souza et al. 2015).

Methods based on DNA polymorphism analysis are used for identification and differentiation of bacteria. They include, *inter alia*, restriction fragment length polymorphism (RFLP) analysis, rep-PCR DNA fingerprinting techniques (repetitive sequence–based PCR), and AFLP (amplified fragment length polymorphisms) (Thies et al. 2001; Albuquerquea et al. 2009).

rep-PCR techniques (ERIC-PCR, BOX-PCR, and REP-PCR) facilitate differentiation of strains even within a single species. The BOX-PCR method is a useful tool in distinguishing and identifying bacteria (Gillings and Holley 1997; Rademaker et al. 2000; Thies et al. 2001; Brusetti et al. 2008). Our results based on the BOX-PCR technique demonstrated wide genomic variation of the *O. arvensis* microsymbionts. It should also be noted that the use of this method may be useful in rapid identification of field restharrow strains.

Analysis of 16S rRNA gene sequences has been widely used for determining the phylogenetic relationship and taxonomic position (mainly genus) of bacterial strains. However, in recent years, it has been found that the resolving power of this technique is limited and it does not allow differentiation of closely related species or even some closely related genera (Rajendhran and Gunasekaran 2011). It has been established that two organisms that have less than 97% sequence similarity of 16S rRNA gene belong to two different species, whereas those with at least 95% 16S rRNA gene sequence similarity belong to the same genus (Rosselló-Mora and Amann 2001; Rajendhran and Gunasekaran 2011).

The phylogenetic analysis based on 16S rRNA gene sequence analysis of seven *O. arvensis* strains (OA4, OA118, OA121, OA129, OA132, OA191 and OA245), representing two genomic groups obtained with the BOX-PCR method showed that rhizobia specific for the restharrow are a phylogenetically diverse group of microorganisms and represent two genera *Rhizobium* and *Mesorhizobium*. Four of them, OA4, OA121, OA129, and OA2445, were classified to the genus *Mesorhizobium*. The remaining three microsymbionts, OA118, OA132, and OA191, formed a common monophyletic cluster with *R. leguminosarum* strains and were affiliated to the genus *Rhizobium*.

Similarly, isolates from root nodules of *Ononis tridentata* L., a plant belonging to the same genus as *O. arvensis*, showed (based on 16S rRNA gene sequence analysis) a phylogenetic relationship with bacteria of the genera *Rhizobium* and *Mesorhizobium*, but some of them also with *Phylobacterium* and *Bosea* (Rincón et al. 2008).

The rhizobial *nod* and *nif* genes are symbiotic, adaptive genes. Some studies suggest that they very often have evolutionary history independent of the housekeeping genes explained by a lateral transfer of *nod* loci even across divergent chromosomal lineages, as in the case of rhizobia representing the genus *Rhizobium* and *Ensifer* (Wang et al. 2007). Horizontal transfer of nodulation genes may adapt rhizobia to a new host plant and enable bacteria with a different genomic background but similar *nod* genes to enter symbiotic interactions with the same legume plants. Some studies of genus *Mesorhizobium* strains suggest that the broad host range of these bacteria may also be the result of the convergence of distinct *nod* genotypes into the same nodulation phenotype (Haukka et al. 1998; Wernegreen and Riley 1999). It is worth noting that currently the *nodC* gene sequence analysis is commonly used for grouping rhizobia into symbiovars (Laguerre et al. 2001; Diouf et al. 2010).

The *nodC* gene sequence analysis of the *O. arvensis* nodule isolates indicated that four strains, OA4, OA121, OA129, and OA245, are phylogenetically closely related to the species *M. tarimense* and *M. loti* (98–99% sequence similarities). The three other isolates from *O. arvensis* nodules, i.e., OA118, OA132, and OA191, as in the 16S rRNA phylogram, grouped with strains of the genus *Rhizobium* (76–97% sequence similarity). It is important to emphasize that the *nodC* gene encoding *N*-acetylglucosaminyltransferase and determining the length of the chitin oligosaccharide chain of the Nod factor is one of the factors associated with the host range (Perret et al. 2000). Interestingly, it was observed that the photosynthetic *Bradyrhizobium* ORS278 and BTAi1 strains forming symbiosis with *Aeschynomene* species did not harbour *nodABC* genes but were still able to establish symbiosis with legume plants. This indicates the existence of independence from Nod factor determinants in rhizobium-legume symbiotic interactions (Wernegreen and Riley 1999; Laguerre et al. 2001; Giraud et al. 2007; Diouf et al. 2010; Okazaki et al. 2016).

All the seven *O. arvensis* microsymbionts studied (OA4, OA118, OA121, OA129, OA132, OA191, and OA245) have been found to possess *acdS* gene sequences very similar to those of *M. loti* NZP2213 and *Mesorhizobium tarimense* CCBAU83306. This might suggest that the OA118, OA132, and OA191 strains, classified to the genus *Rhizobium* by the 16S rRNA gene sequence analysis, have acquired the *acdS* gene through horizontal transfer from *Mesorhizobium* bacteria. The acquisition of the *acdS* gene by lateral transfer of a symbiotic island has been demonstrated in some *Mesorhizobium* sp. strains (Nandasena et al. 2007; Glick 2014; Nascimento et al. 2014).

The host range is still an important feature in the description of new species of nodule bacteria (Graham et al. 1991). Up to now, there has been little information available about the host range of *O. arvensis* microsymbionts. Novikova et al. (1993) described five strains isolated from *O. arvensis* root nodules that formed symbiosis also with *L. corniculatus*. The results of our plant tests demonstrated that bacteria specific for *O. arvensis* exhibit a narrow host range and induce effective nitrogen fixing nodules only on the roots of their host plant and *O. spinosa* but not on *T. repens*, *M. sativa* or *L. corniculatus*. The ability of restharrow symbionts to form mutualistic interactions with *O. spinosa*, i.e. a plant which is under partial protection in Poland, can be useful in protecting and promoting the growth of this fabacean.

Fabacean plants, similar as nodule bacteria, differ in their symbiotic specificity and one plant species may interact with many taxonomically different rhizobia species, whereas another one forms symbiosis with unique root nodule bacteria (Ferro et al. 2000; Laranjo et al. 2014). In the plant tests, *O. arvensis* was effectively nodulated by many *Mesorhizobium* and *Rhizobium* species but not by *Ensifer, Neorhizobium*, and *Bradyrhizobium* species and therefore, *O. arvensis* may be determined as a promiscuous host for rhizobia.
